# Smoking as an independent risk factor for hepatocellular carcinoma: the Singapore Chinese Health Study

**DOI:** 10.1038/bjc.2011.360

**Published:** 2011-09-13

**Authors:** W-P Koh, K Robien, R Wang, S Govindarajan, J-M Yuan, M C Yu

**Affiliations:** 1 Department of Epidemiology and Public Health, Yong Loo Lin School of Medicine, National University of Singapore, MD3, 16 Medical Drive, Singapore 117597, Singapore; 2 Division of Epidemiology and Community Health, School of Public Health, University of Minnesota, Minneapolis, MN, USA; 3 The Masonic Cancer Center, University of Minnesota, Minneapolis, MN, USA; 4 Department of Clinical Pathology, Rancho Los Amigos National Rehabilitation Center, Keck School of Medicine, University of Southern California, Los Angeles, CA, USA

**Keywords:** smoking, hepatocellular carcinoma, alcohol drinking, viral hepatitis, prospective cohort, Chinese

## Abstract

**Background:**

Given the close correlation between smoking and alcohol intake in most epidemiologic studies, it is difficult to exclude the residual confounding effect of alcohol in the association between smoking and hepatocellular carcinoma (HCC).

**Method:**

We evaluated the association between smoking and risk of HCC in the Singapore Chinese Health Study, a prospective cohort with a low prevalence of alcohol intake. Information on cigarette smoking and alcohol consumption was obtained through in-person interviews conducted at enrolment.

**Results:**

After a mean of 11.5 years of follow-up, there were 394 incident cases of HCC. Participants who consumed more than two alcoholic drinks per day showed an increased risk for HCC (hazard ratio (HR)=2.24; 95% confidence interval (CI)=1.46–3.41). After adjusting for alcohol consumption and other potential confounders, current *vs* never smokers had a statistically significant, increased risk of HCC (HR=1.63; 95% CI=1.27–2.10) that was dose-dependent (number of cigarettes per day, *P* for trend<0.001). The observed tobacco–HCC association also was duration-dependent (years of smoking in ever smokers, *P* for trend=0.002). When we excluded daily drinkers from the analysis, all risk estimates remained essentially the same and statistically significant.

**Conclusion:**

Our findings strongly implicate tobacco smoke as a causal factor of HCC development.

Although the incidence rates of hepatocellular carcinoma (HCC) may vary by as much as 40-fold worldwide, the causal factors accounting for the highest population attributable risk in high-risk and low-risk populations are also different (Yu *et al*, 2000; Kao and Chen, 2002; Parkin *et al*, 2002). In high-risk populations such as those in China and Africa, chronic infection from hepatitis B virus (HBV) is the most important risk factor (Yeh *et al*, 1989; Kew *et al*, 1997). Conversely, in low-risk countries in the west, such as the United States, chronic alcohol abuse is an important contributor (Yu and Yuan, 2004; Yuan *et al*, 2004). Unlike HBV infection and excess alcohol intake, which are well recognised as risk factors of HCC, the contribution of cigarette smoking to HCC risk is less well established. Although cigarette smoking has also been studied in association with HCC risk in case–control and cohort studies conducted in diverse populations (reviewed by Lee *et al*, 2009), the validity of such results is often in question. In populations where excessive alcohol intake is a strong risk factor, cigarette smoking and alcohol intake are often too closely correlated to rule out residual confounding effect of alcohol in the examination of smoking as an independent risk factor for HCC (Thornton *et al*, 1994; Brasche *et al*, 1998; Ma *et al*, 2000; Koh *et al*, 2005). Similarly, in populations, which are endemic for HBV infection, it may also be difficult to rule out HBV infection as a confounding factor. In a recent meta-analysis of 38 cohort studies, only 6 studies from two populations (four in Japan and two in Taiwan) included both HBV and alcohol consumption as potential confounders in the examination of smoking as a risk factor of HCC (Lee *et al*, 2009). Hence, despite evidence from experimental studies that constituents of tobacco smoke may be hepatic carcinogens (IARC, 1978, 1987; Dooley *et al*, 1992), it remains unclear whether observed associations between smoking and HCC risk in human populations (Trichopoulos *et al*, 1987; Hsing *et al*, 1990; Chiba *et al*, 1996; Yuan *et al*, 2004) are due directly to exposure to tobacco smoke, or indirectly through alcohol intake or HBV infection.

In Singapore, HCC is one of the top five cancers among men with an age-standardised incidence of 17.4 per 100 000 person-years in 2008 (National Registry of Diseases Office, 2010), although the rate is lower than those noted in high-risk regions of China, including Southern Guangxi and Shanghai (Seow *et al*, 2004). The Singapore Chinese Health Study is a cohort with a wide range of smoking duration and dosage, but practically nil excessive alcohol intake (usually defined as four or more drinks per day) against a background of a very low prevalence (11.7%) of weekly or more frequent intake of alcohol (the standard definition of regular alcohol intake) (Koh *et al*, 2005). Thus, we aimed to conduct a definitive examination of the independent effect of smoking on HCC risk, in the absence of any confounding effects of alcohol intake, in this cohort.

## Materials and methods

The design of the Singapore Chinese Health Study has been described (Hankin *et al*, 2001). Briefly, the cohort of 63 257 men and women, aged 45–74 years, was recruited between April 1993 and December 1998 from residents of Singapore who resided in government-built housing estates, and belonging to the two major dialect groups (Hokkiens and Cantonese) of Chinese in Singapore. The present study included the 61 321 cohort subjects who were free of a history of invasive cancer at enrolment. All participants gave informed consent, and the study was approved by the Institutional Review Boards of the National University of Singapore and the University of Minnesota.

At recruitment, an in-person interview was conducted using a structured questionnaire, which included a 165-item food frequency questionnaire, that was subsequently validated against a series of 24-h dietary recall interviews (Hankin *et al*, 2001) conducted on a random subset of cohort participants. Alcohol drinkers were defined as individuals who drank any alcoholic beverage on a monthly basis or more often. One drink was defined as 375 ml of beer (13.6 g of ethanol), 118 ml of wine (11.7 g of ethanol), or 30 ml of western or Chinese hard liquor (10.9 g of ethanol). Total number of drinks and grams of ethanol consumed per day were computed based on the amount and type of alcoholic beverages consumed. For cigarette smoking, the subjects were asked, ‘Have you ever smoked at least one cigarette a day for 1 year or longer’ and defined as ‘never-smokers’ for those who answered ‘no’, ‘former smokers’ for those who answered ‘yes, but I quit smoking’, and ‘current smokers’ for those who answered ‘yes and I currently smoke’. Ever smokers were then asked about number of cigarettes smoked per day (six predetermined categories: six or less, 7–12, 13–22, 23–32, 33–42, and 43 or more) and number of years of smoking (four pre-determined categories: <10, 10–19, 20–39, 40+).

As on 31 December 2007, 394 incident cases of HCC were identified through linkage with the population-based Singapore Cancer Registry. Almost all HCC cases (96.9%) were diagnosed histologically and their diagnoses were confirmed via manual review of pathology reports by a medically trained research staff. We constructed a case–control study nested within this cohort using the following criteria for the selection of cases and controls. Cases were all subjects who developed HCC after they donated a blood sample. For each case, three control subjects were randomly chosen among all the cohort participants who were alive and free of any cancer at the time of cancer diagnosis of their respective index case. The controls were individually matched to their index cases on year at recruitment (±1 year), year of birth (±2 years), gender, dialect group (Cantonese, Hokkien), and date of biospecimen collection (±6 months). Ninety-two HCC cases and 274 individually matched control subjects who met the above criteria were included in the present analysis. Their sera were tested for the presence of hepatitis B surface antigen (HBsAg, indicative of chronic carrier status), antibodies to HBsAg (anti-HBs, indicative of immunity to HBV infection resulting from natural exposure to HBV or HBV vaccine), antibodies to hepatitis B core antigen (anti-HBc, indicative of a history of primary infection), and antibodies to HCV (anti-HCV, indicative of a history of hepatitis C infection).

### Laboratory tests

We collected blood and urine specimens from a random 3% sample of study enrollees, beginning in April 1994, and that was extended to all surviving cohort members, beginning in January 2000. At completion (April 2005), biospecimens were obtained from 32 543 subjects, representing a consent rate of 60% in contacted subjects. Details of the biospecimen collection, processing, and storage procedures have been described previously (Koh *et al*, 2003).

Blood samples from cases and controls were processed and stored (−20 °C) in an identical manner. The assays used for testing serologic markers of HBV and HCV infections have been described previously (Johnson *et al*, 2011). Briefly, we tested all study samples for the presence of HBsAg using commercialised kits (AUSRIA, Abbott Laboratories, North Chicago, IL, USA), and negative samples were further tested for the presence of anti-HBc and anti-HBs using standard testing kits (Corab and Ausab, respectively, Abbott Laboratories). All samples were tested for the presence of anti-HCV using the ELISA version 2.0 kit manufactured by Ortho Diagnostic Systems (a Johnson & Johnson company, Raritan, NJ, USA), with confirmation of positive samples using the RIBA version 2.0 (Chiron, Emeryville, CA, USA). Serum samples were tested blindly, identified only by codes without regard to the case/control status.

### Statistical analysis

Cox proportional hazards regression methods were used to examine the association between cigarette smoking and HCC risk within the entire cohort. The magnitude of the associations was assessed by the hazard ratios (HRs) and their corresponding 95% confidence intervals (CIs), and *P*-values. All Cox regression models included the following covariates: age at recruitment (year), gender, dialect group (Hokkien, Cantonese), year of recruitment (1993–1995, 1996–1998), the level of education (no formal education, primary school, secondary school or higher), body mass index (<20, 20–24, 24–28, 28+ kg m^−2^), history of diabetes (yes, no), and coffee drinking (<2, 2–3, and 3+ cups per day). The latter three covariates were included as they have been established as risk factors either in this cohort (Johnson *et al*, 2011) or in other studies (Bravi *et al*, 2007; Noto *et al*, 2010; Saunders *et al*, 2010).

The Fisher's exact test was used to compare the distribution of cigarette smoking by history of chronic viral hepatitis infection (positivity for HBV and/or HCV) among the controls of the nested case–control study. Conditional logistic regression methods were used to examine the relationships between markers of HBV and HCV infections and HCC risk within the nested case–control set. Unconditional logistic regression models used to examine the association between smoking and HCC risk in this nested case–control set included age at recruitment (year), gender, dialect group (Hokkien, Cantonese), year of recruitment (1993–1995, 1996–1998), the level of education (no formal education, primary school, secondary school or higher), daily ethanol intake (grams), body mass index (<20, 20–24, 24–28, 28+ kg m^−2^), history of diabetes (yes, no), and coffee drinking (<2, 2–3, and 3+ cups per day). The magnitude of the associations was assessed by the odds ratios (ORs) and their corresponding 95% CIs and *P*-values. Results were not statistically different between men and women. Therefore, all figures presented are based on total subjects. Statistical computing was conducted using the SAS version 9.1 (SAS Institute Inc., Cary, NC, USA) statistical software package. All *P*-values quoted were two-sided. *P*-values of <0.05 were considered statistically significant.

## Results

Table 1 shows the association between HBV/HCV serology and risk of HCC among the nested case–control set of 92 cases and 274 controls. The mean (±s.d.) age of cases was 68.9 (8.6) years at diagnosis, which was comparable with that of controls (69.0±8.3). The average time intervals between baseline interview and cancer diagnosis were 8.8 (±2.9) years for cases and 8.8 (±2.9) years for controls. Men accounted for 75% of both cases and controls, respectively. Among the 92 HCC cases, 25.0% were current smokers and 18.5% were former smokers. The corresponding figures for controls were 24.8 and 19.7%. The smoking intensity and duration between cases and controls were comparable. Cases were more likely to be daily drinkers (7.5% among cases *vs* 4.4% among controls) and to have a history of diabetes (22.8% among cases *vs* 9.1% among controls). One-third cases and one-fourth controls drank either green tea or black tea daily, while two-third cases and three-fourth controls consumed at least one cup of coffee a day.

Chronic carriers of HBV (HbsAg-positive subjects) showed the highest risk of HCC (OR=24.79; 95% CI=8.61–71.34). Our data suggest that subjects with a history of primary infection of HBV (anti-HBc positive) but absence of subsequent immunity (anti-HBs negative) might also be at an increased risk of HCC, although the risk level was an order of magnitude lower than those positive for HBsAg (OR=2.01; 95% CI=0.92–4.39). On the other hand, subjects showing presence of serum anti-HBs were not at an increased risk for HCC. HCV infection was rare in this population. Only five HCC cases (5%) and three control subjects (1%) were positive for anti-HCV. Nevertheless, HCV infection was statistically significantly related to HCC (OR=10.12; 95% CI=2.19–46.80).

Table 2 shows the association between alcohol consumption and HCC risk in the entire cohort. Relative to non-drinkers of alcohol, moderate drinkers of up to two drinks daily did not show an association with HCC risk. On the other hand, consuming more than two alcoholic drinks per day was associated with a statistically significant, two-fold risk of HCC (HR=2.24; 95% CI=1.46–3.41).

Table 3 presents the association between cigarette smoking and HCC risk, separately for all the cohort subjects and with the exclusion of daily alcohol drinkers who were shown in Table 2 to show an increased risk for HCC. After adjusting for alcohol consumption and other potential confounders, current *vs* never smokers had a statistically significant, increased risk of HCC (HR=1.63; 95% CI=1.27–2.10). Current smokers also had a statistically significant and dose-dependent association between number of cigarettes smoked per day and HCC risk. Among ever smokers, duration and pack-years of smoking were associated with HCC risk in a statistically significant, dose-dependent manner. Results remained essentially the same when daily alcohol drinkers were excluded from the analysis (Table 3).

In this study population, among the control subjects in Table 1, the prevalence of cigarette smoking was not statistically different by history of chronic viral hepatitis infection (positivity for HBV and/or HCV). Among controls with viral hepatitis history, 51% were ever smokers compared with 43% among those without such a history (*P*-value for Fisher's exact test=0.36).

We further examined the cigarette smoking–HCC association within the nested case–control set of cohort subjects with HBV/HCV serology measurements. Specifically, we aimed to investigate the association between tobacco smoking and HCC risk in the absence of viral hepatitis infections and daily alcohol intake (Table 4). HBV positivity was defined as being HBsAg positive or anti-HBc positive, but anti-HBs negative; HCV positivity was defined as being anti-HCV positive. There were only 38 HCC cases and 221 control subjects who were both HBV and HCV negative, rendering the sample size too small to be meaningfully analysed for statistical significance. Nevertheless, it is worth noting that ever smoking was associated with a 1.6-fold increase in HCC risk (OR=1.60; 95% CI=0.61–4.21) in this subset. When we further excluded subjects who were daily drinkers from this subset of subjects who were negative for both HBV and HCV, the risk associated with ever smoking was 1.85-fold that of never smokers (HR=1.85; 95% CI=0.66–5.18).

## Discussion

In this study conducted among the Singapore Chinese, a population possessing a high risk for HCC but a very low prevalence of alcohol consumption, we have provided the strongest epidemiologic evidence to date that smoking is a causal factor for HCC development. Specifically, our results show a statistically significant, dose- and duration-dependent association between tobacco use and HCC risk that is absent of possible residual confounding effects of alcohol consumption or chronic viral hepatitis infection on tobacco smoking.

Chronic alcohol abuse is a well-established aetiologic factor of HCC and is a factor of high attributable risk, especially in developed countries of North America and Western Europe (IARC, 1988). A recent meta-analysis of 10 studies suggests that significant increased risk of HCC was associated with an ethanol intake of 25 g per day or the equivalent of two drinks per day, which was the lowest dose of alcohol considered (Corrao *et al*, 2004). Similarly, we also documented an increased HCC risk only among daily drinkers of more than two drinks per day.

Prospective data on smoking and HCC risk remain controversial. Although two cohorts of Asian men in Japan and Taiwan reported positive does–response associations between smoking and HCC risk (Hirayama, 1989; Chen *et al*, 1993), another cohort study in Japan did not find the increased risk of HCC in smokers to remain significant after adjusting for age and gender (Mori *et al*, 2000). Other cohort studies that have found an association between smoking and HCC risk also either only studied men (Wang *et al*, 2003; Yun *et al*, 2005) or did not find this association among women (Goodman *et al*, 1995; Jee *et al*, 2004). It is recognised that smoking and alcohol drinking are highly correlated lifestyle factors, independent of geography and race–ethnicity (Thornton *et al*, 1994; Brasche *et al*, 1998; Ma *et al*, 2000; Koh *et al*, 2005). Hence, in a study population with a high prevalence of regular (weekly or daily) alcohol intake, residual confounding can never be ruled out as an alternative explanation for any observed tobacco–HCC association.

A unique strength of this study is therefore the very low prevalence (11.7% of weekly or daily use) of regular alcohol intake among the study subjects. We and others have shown that moderate daily consumption of alcohol (under two drinks per day) is unrelated to HCC risk and that sustained consumption of two or more drinks per day is required for an observed increased risk of HCC (Bagnardi *et al*, 2001; Yu and Yuan, 2004; Yuan *et al*, 2004). Only 1463 cohort subjects (2.4%) and 24 HCC cases (6.1%) consumed this high amount of daily alcohol. Furthermore, all observed associations between tobacco smoking and HCC risk remained statistically significant, and dose- and duration-dependent upon exclusion of all daily alcohol drinkers from the analysis.

This report also presents the first prospective delineation of the quantitative relationship between serological markers of HBV and HCV infections and HCC risk among the Chinese in Southeast Asia. Prevalence of positivity for HBsAg, a marker of chronic carrier state, was 39% among the cases, and risk of HCC among HbsAg-positive subjects was 25 times that among negative subjects. Chronic infections with HBV has been established as the most important attributable factor for HCC among the Chinese in mainland China (Yeh *et al*, 1989) and Taiwan (Wang *et al*, 2003), and our study shows this also to be the situation for the Chinese in Singapore. The prevalence of 2.9% for HBsAg positivity and 46.7% seropositivity for anti-HBs among the controls was comparable to previously reported prevalence from national surveys in Singapore (Hong *et al*, 2010). In our study, presence of anti-HBc (a marker of past primary infection) without subsequent development of immunity (anti-HBs negative) was associated with a non-statistically significant two times increased risk of HCC, and this serologic marker has been shown to predict HCC risk in low-risk populations for both HBV infection and HCC, although the associated risk estimate is of an order of magnitude lower than that in HbsAg-positive chronic carriers (Yu *et al*, 1997). Similar to the situation in mainland China (Yu *et al*, 2000), hepatitis C infection has a negligible role in the development of HCC among the Singapore Chinese due to its low prevalence.

In this study as well as the one conducted in Taiwan (Wang *et al*, 2003), non-carriers of HBV had a lower prevalence of smoking than carriers. Hence, HBV infection can potentially confound the smoking–HCC association. In a study in Italy, the smoking–HCC risk association was only observed in HBV carriers (Franceschi *et al*, 2006). In the present study, smokers free of HBV/HCV infections and who did not consume alcohol on a daily basis were shown to exhibit a 1.85-fold increased risk of HCC.

Other strengths of the present study are its population-based design, a large study sample size, prospective data obtained via a face-to-face interview, and near-complete cancer case ascertainment through a comprehensive nationwide cancer registry (Parkin *et al*, 2002) in a small city–state with a system for easy access to specialized medical care. Other possible confounders such as body mass index, history of diabetes mellitus, and coffee consumption (included in the regression models) were assessed before cancer diagnosis and thus can be presumed to be free of recall bias. A potential limitation of this study is the possible change in the habits of smoking and alcohol use in cohort subjects post enrolment. A comparison of responses obtained during our Follow-up I Survey (Butler *et al*, 2006), which was conducted, on average, about 6 years post enrolment, revealed that only 0.9% of non- or occasional alcohol drinkers at baseline self-reported as daily drinkers at Follow-up I, whereas 32.7% of daily drinkers at baseline self-reported as non- or occasional drinkers at Follow-up I. Similarly, only 18.7% of current smokers at baseline self-reported as ex-smokers at Follow-up I, whereas 9.1% of ex-smokers at baseline self-reported as current smokers at Follow-up I.

There are ample experimental data in support of a causal link between tobacco smoking and HCC development. The liver is a major organ for the metabolism and transformation of more than 40 tobacco-related active compounds (Hoffmann *et al*, 1993), several of which are well-accepted carcinogens such as polycyclic aromatic hydrocarbons, nitrosamines, and aromatic amines (Staretz *et al*, 1997; Chen *et al*, 2002). Several tobacco-related carcinogens, such as *N-*nitrosodimethylamine and 4-aminobiphenyl, have been directly implicated in the development of liver tumours in animal studies (IARC, 1978, 1987; Dooley *et al*, 1992).

In conclusion, this study has provided the strongest epidemiologic evidence to date that tobacco smoking is a causal factor for HCC development in humans.

## Figures and Tables

**Table 1:** HBV and HCV serology in relation to risk of hepatocellular carcinoma: the Singapore Chinese Health Study

**HBV/HCV serology**	**Cases (*n*=92)^a^**	**Controls (*n*=274)^a^**	**Odds ratios (95% CI)^b^**
Negative on all four markers	17	95	1.00
Anti-HBs positive	22	128	1.10 (0.54–2.22)
HBsAg positive	36	8	24.79 (8.61–71.34)
Anti-HBc positive, but both HBsAg and anti-HBs negative	16	43	2.01 (0.92–4.39)
HBsAg positive or anti-HBc positive, but anti-HBs negative (HBV positive)	52	51	5.34 (2.44–11.67)
Anti-HCV positive	5	3	10.12 (2.19–46.80)

Abbreviations: anti-HBc=antibodies to hepatitis B core antigen; anti-HBs=antibodies to hepatitis B surface antigen; anti-HCV=antibodies to hepatitis C virus; CI=confidence interval; HbsAg=hepatitis B surface antigen; HBV=hepatitis B virus; HCV=hepatitis C virus.

a The sum of cases and controls across all categories of HBV/HCV serology was greater than the total number of subjects, as these serology groups were not mutually exclusive.

b Odds ratios were calculated using conditional logistic regression models with further adjustment for level of education (no formal education, primary, secondary or higher).

**Table 2:** Alcohol intake in relation to risk of hepatocellular carcinoma: the Singapore Chinese Health Study

**Alcohol intake**	**Cases**	**HR (95% CI)^a^**	**HR (95% CI)^b^**
Non-drinkers	308	1.00	1.00
Less than daily	55	0.76 (0.57–1.02)	0.79 (0.59–1.06)
			
*Daily drinkers*	31	1.58 (1.09–2.30)	1.75 (1.20–2.54)
Up to two drinks per day	7	0.87 (0.41–1.84)	1.01 (0.48–2.14)
More than two drinks per day	24	2.09 (1.37–3.19)	2.24 (1.46–3.41)

Abbreviations: CI=confidence interval; HR=hazards ratio.

a Hazard ratios were adjusted for gender, age at recruitment (years), year of recruitment, dialect group (Hokkien, Cantonese), and the level of education (no formal education, primary, secondary or higher).

b Hazard ratios were further adjusted for body mass index (<20, 20–24, 24–28+ kg m^−2^), diabetes mellitus (yes, no), and cups of coffee per day (<2 cups, 2–3 cups, 3+ cups).

**Table 3:** Cigarette smoking in relation to risk of hepatocellular carcinoma: the Singapore Chinese Health Study

	**Total**		**Excluding daily drinkers**	
	**Cases**	**HR (95% CI)^a^**	**Cases**	**HR (95% CI)^a^**
*Smoking status*				
Never smoker	185	1.00	181	1.00
Former smoker	72	1.13 (0.84–1.51)	67	1.12 (0.83–1.52)
Current smoker	137	1.63 (1.27–2.10)	115	1.56 (1.20–2.04)
				
*Number of cigarettes per day among current smokers*				
1–12 cigarettes per day	51	1.53 (1.10–2.12)	46	1.49 (1.06–2.09)
13+ cigarettes per day	86	1.72 (1.28–2.30)	69	1.63 (1.19–2.23)
*P* for trend^b^		<0.001		<0.001
				
*Number of years of smoking*				
Never smoker	185	1.00	181	1.00
<20	22	1.13 (0.72–1.78)	20	1.09 (0.68–1.76)
20+	187	1.46 (1.15–1.85)	162	1.41 (1.11–1.81)
*P* for trend		0.002		0.006
				
*Number of pack-years of smoking*				
Never smoker	185	1.00	181	1.00
<20	55	1.21 (0.88–1.65)	50	1.17 (0.84–1.62)
20–<40	107	1.53 (1.17–2.00)	96	1.53 (1.15–2.02)
40+	47	1.51 (1.06–2.15)	36	1.37 (0.93–2.01)
*P* for trend		0.002		0.007

Abbreviations: CI=confidence interval; HR=hazards ratio.

a Hazard ratios were adjusted for gender (for total sample), age at recruitment (years), year of recruitment, gender (for total sample), dialect group (Hokkien, Cantonese), the level of education (no formal education, primary, secondary or higher), body mass index (<20, 20–24, 24–28+ kg m^−2^), diabetes mellitus (yes, no), daily ethanol intake (in grams), and cups of coffee per day (<2 cups, 2–3 cups, 3+ cups) for total subjects.

b Trend test is based on the four ordinal levels of smoking: never smoker, former smoker, current/1–12 cigarettes per day smoker, and current/13+ cigarettes per day smoker.

**Table 4:** Cigarette smoking in relation to risk of hepatocellular carcinoma among hepatitis serology negative (HBV and HCV negative) subjects: the Singapore Chinese Health Study

	**Never smokers**		**Ever smokers**	
**HBV and HCV negative**	**Cases/controls**	**OR**	**Cases/controls**	**OR (95% CI)^a^**
All	20/126	1.00	18/95	1.60 (0.61–4.21)
Drank alcohol less than weekly	18/116	1.00	12/75	1.82 (0.59–5.66)
Drank alcohol less than daily	19/126	1.00	17/88	1.85 (0.66–5.18)

Abbreviation: CI=confidence interval.

a Odds ratios (OR) were calculated using unconditional logistic regression models with adjustment for age at recruitment (years), gender, dialect group (Hokkien, Cantonese), level of education (no formal education, primary, secondary or higher), body mass index (<20, 20–24, 24–28+ kg m^−2^), diabetes mellitus (yes, no), daily ethanol intake (grams), and cups of coffee per day (<2 cups, 2–3 cups, 3+ cups).
